# Supplementary material for: Accurate predictions of individual differences in task-evoked brain activity from resting-state fMRI using a sparse ensemble learner
Source: Neuroimage. 2022 Oct 1;259:119418. doi: 10.1016/j.neuroimage.2022.119418 (PMC10933828; doi:10.1016/j.neuroimage.2022.119418)
Supplement: Supplementary Data S1 — Supplementary Raw Research Data. This is open data under the CC BY license http://creativecommons.org/licenses/by/4.0/ [file mmc1.pdf]

SUPPLEMENTARY INFORMATION

**HCP contrast category**	**HCP contrast index and name**
EMOTION	01_EMOTION_FACES 02_EMOTION_SHAPES 03_EMOTION_FACES-SHAPES
GAMBLING	07_GAMBLING_PUNISH 08_GAMBLING_REWARD 09_GAMBLING_PUNISH-REWARD
LANGUAGE	13_LANGUAGE_MATH 14_LANGUAGE_STORY 15_LANGUAGE_MATH-STORY
MOTION	19_MOTOR_CUE 20_MOTOR_LF 21_MOTOR_LH 22_MOTOR_RF 23_MOTOR_RH 24_MOTOR_T 25_MOTOR_AVG 26_MOTOR_CUE-AVG 27_MOTOR_LF-AVG 28_MOTOR_LH-AVG 29_MOTOR_RF-AVG 30_MOTOR_RH-AVG 31_MOTOR_T-AVG
RELATIONAL	45_RELATIONAL_MATCH 46_RELATIONAL_REL 47_RELATIONAL_MATCH-REL
SOCIAL	51_SOCIAL_RANDOM 52_SOCIAL_TOM 53_SOCIAL_RANDOM-TOM
WORKING MEMORY	57_WM_2BK 58_WM_2BK 59_WM_2BK 60_WM_2BK 61_WM_0BK 62_WM_0BK 63_WM_0BK 64_WM_0BK 65_WM_2BK 66_WM_0BK 67_WM_2BK-0BK 71_WM_BODY 72_WM_FACE 73_WM_PLACE 74_WM_TOOL 75_WM_BODY-AVG 76_WM_FACE-AVG 77_WM_PLACE-AVG 78_WM_TOOL-AVG

TABLE S1. List of the 47 HCP contrasts. We used the 47 unique contrast maps for HCP, excluding all redundant contrasts.

Notation	Explanation	Notation	Explanation
V	the number of voxels	k	the number of dual-regression maps per subject
$\mathcal{S}$	the training set	$\mathcal{T}$	the test set
N	the number of training subjects	n	the number of test subjects
$\mathbf{x}_j^i \in \mathbb{R}^V$	the i-th resting-state variation map of subject j	$\mathbf{X}_j \in \mathbb{R}^{V\times k}$	the resting-state variation maps of subject j
$\boldsymbol{\beta}_j, \hat{\boldsymbol{\beta}}_j \in \mathbb{R}^k$	the (estimated) baseline coefficients for subject j	$\hat{\boldsymbol{\beta}} \in \mathbb{R}^k$	the baseline coefficients (averaged across the training set)
$\tilde{\mathbf{X}}_{\mathcal{S}}^i \in \mathbb{R}^{N\times V}$	the i-th across-subject resting-state variation matrix (of the training set)	$\mathbf{Y}_{\mathcal{S}}, \hat{\mathbf{Y}}_{\mathcal{S}} \in \mathbb{R}^k$	the (predicted) across-subject task variation matrix
$\mathbf{A}_{\mathcal{S}}^i, \hat{\mathbf{A}}_{\mathcal{S}}^i \in \mathbb{R}^{N\times d}$	the (estimated) mixing matrix of the i-th across-subject resting-state variation matrix	$\mathbf{S}^i \in \mathbb{R}^{d\times V}$	the independent components of the i-th across-subject resting-state variation matrix
$\mathbf{A}_{\mathcal{S}}^{\text{rest}}, \hat{\mathbf{A}}_{\mathcal{S}}^{\text{rest}} \in \mathbb{R}^{N\times dk}$	the concatenated k mixing matrices of the resting-state variation matrices	d	the number of modes/independent components of each resting-state variation matrix
$\mathbf{A}_{\mathcal{S}}^{\text{task}} \in \mathbb{R}^{N\times p}$	the mixing matrix of the across-subject task variation matrix	$\mathbf{S}^{\text{task}} \in \mathbb{R}^{p\times V}$	the independent components of the task variation matrix
$\mathbf{y}_j \in \mathbb{R}^V$	the task variation map of subject j	p	the number of modes/independent components of the task variation matrix
$\mathbf{W}, \hat{\mathbf{W}} \in \mathbb{R}^{dk\times p}$ or $\mathbb{R}^{dk\times V}$	the (estimated) sparse coefficients	$\mathbf{w}_i, \hat{\mathbf{w}}_i \in \mathbb{R}^{dk}$	the i-th column of the estimated sparse coefficients
λ_i	the hyper-parameter of the L_1 penalty for the i-th column of $\mathbf{W}$	$\hat{\mathbf{Y}}_{\mathcal{S}}^{\text{baseline}} \in \mathbb{R}^{N\times V}$	the baseline-model-fitted task variation matrix for the training subjects
$\hat{\mathbf{Y}}_{\mathcal{S}}^{\text{sparse}} \in \mathbb{R}^{N\times V}$	the sparse-model-fitted task variation matrix for the training subjects	$\hat{\mathbf{y}}_{\cdot i}^{\text{baseline}}, \hat{\mathbf{y}}_{\cdot i)}^{\text{sparse}} \in \mathbb{R}^N$	the i-th column/voxel of the baseline- / sparse-model fitted task variation matrix
$\theta_i^{(1)}$	ensemble coefficient for the i-th voxel of the baseline mode	$\theta_i^{(2)}$	ensemble coefficient for the i-th voxel of the sparse mode

TABLE S2. List of the notations.

Prediction accuracy of the residualised activations	UKB	HCP
DR-ICA baseline vs PFM baseline	p<1e-30	p<1e-30
DR-ICA sparse vs PFM sparse	p<1e-30	p<1e-30
DR-ICA ensemble vs PFM ensemble	p<1e-30	p<1e-30
DR-ICA baseline vs DR-ICA sparse	p<1e-30	p<1e-30
DR-ICA baseline vs DR-ICA ensemble	p<1e-30	p<1e-30
DR-ICA sparse vs DR-ICA ensemble	p<1e-30	p<1e-30
PFM baseline vs PFM sparse	p<1e-30	p<1e-30
PFM baseline vs PFM ensemble	p<1e-30	p<1e-30
PFM sparse vs PFM ensemble	p<1e-30	p<1e-30

TABLE S3. Two-sided paired t-test between prediction accuracies of the baseline, sparse and ensemble model (residualised), based on DR-ICA or PFM repsectively. The task contrasts were pooled together, corresponding to the last columns of Figure 2a and 2b (UKB three contrasts and HCP 47 contrasts, across all subjects). The p-values were all highly significant, after being Bonferroni-corrected (18 tests in total).

Correlation with inter-individual variability	UKB	HCP
DR-ICA baseline vs PFM baseline	0.0243	4.5176e-12
DR-ICA sparse vs PFM sparse	0.6724	0.0003
DR-ICA ensemble vs PFM ensemble	1.1192	0.0218
DR-ICA baseline vs DR-ICA sparse	0.8775	5.7470e-5
DR-ICA baseline vs DR-ICA ensemble	0.6582	1.4465e-16
DR-ICA sparse vs DR-ICA ensemble	0.1026	1.7612e-25
PFM baseline vs PFM sparse	3.2002	0.1466
PFM baseline vs PFM ensemble	2.3015	5.4127e-8
PFM sparse vs PFM ensemble	0.0005	5.5898e-23

TABLE S4. Two-sided paired t-test between correlations with the inter-individual variability (std map) of the baseline, sparse and ensemble model (residualised), based on DR-ICA or PFM repsectively. The task contrasts were pooled together, corresponding to the last columns of Figure 2c and 2d (UKB three contrasts and HCP 47 contrasts). The p-values were Bonferroni-corrected (18 tests in total). Overall, UKB results were non-significant, which is un-surprising given that UKB has only three task contrasts (i.e., three samples).

Accuracy & discriminability		UKB	HCP
Tavor method vs un-residualised	accuracy	p<1e-30	p<1e-30
	discriminability	p<1e-30	p<1e-30
Tavor method vs residualised	accuracy	p<1e-30	p<1e-30
	discriminability	p<1e-30	p<1e-30
un-residualised method vs residualised	accuracy	0.4105	3.7932
	discriminability	p<1e-30	p<1e-30

TABLE S5. Two-sided paired t-test between model performance of different approaches. The task contrasts were pooled together, corresponding to the last columns of Figure 3 (UKB three contrasts and HCP 47 contrasts, across all subjects). The p-values were Bonferroni-corrected (12 tests in total).

Comparison with test-retest reliability (task prediction)		UKB	HCP
first-visit prediction vs reliability	un-residualised	p<1e-30	p<1e-30
	residualised	p<1e-30	p<1e-30
second-visit prediction vs reliability	un-residualised	p<1e-30	p<1e-30
	residualised	p<1e-30	p<1e-30

TABLE S6. Two-sided paired t-test between model performance (with group-average added back in) and test-retest reliability. The task contrasts were pooled together, corresponding to the last columns of Figure 4a and 4b (UKB three contrasts and HCP 47 contrasts, across all subjects). The p-values were all highly significant, after being Bonferroni-corrected (8 tests in total).

Comparison with test-retest reliability (amplitude prediction)	UKB	HCP
first-visit prediction vs reliability	1.2543	6.5663e-11
second-visit prediction vs reliability	0.0734	2.9706e-6

TABLE S7. Two-sided paired t-test between amplitude prediction accuracy and the corresponding test-retest reliability. The task contrasts were pooled together, corresponding to the last columns of Figure 4c and 4d (UKB three contrasts and HCP 47 contrasts). The p-values were Bonferroni-corrected (4 tests in total). UKB results were non-significant, which is un-surprising given that UKB has only three task contrasts (i.e., three samples).

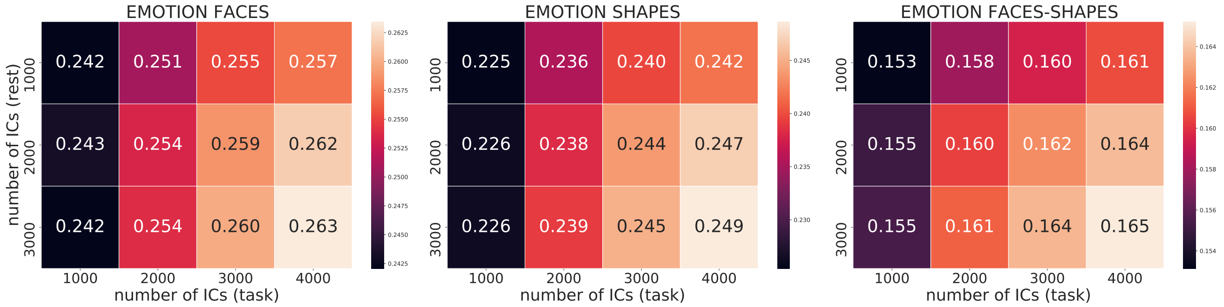

Figure S1. Prediction accuracy of the sparse model at a range of PFM dimensions, trained on a subset of 4,000 UKB subjects and tested on 700. Overall, prediction accuracy increases with the number of functional modes. Note that the results were based on residualised data. The un-residualised data exhibited similar accuracy patterns, though with smaller differences between the choices of dimensions (not shown here).

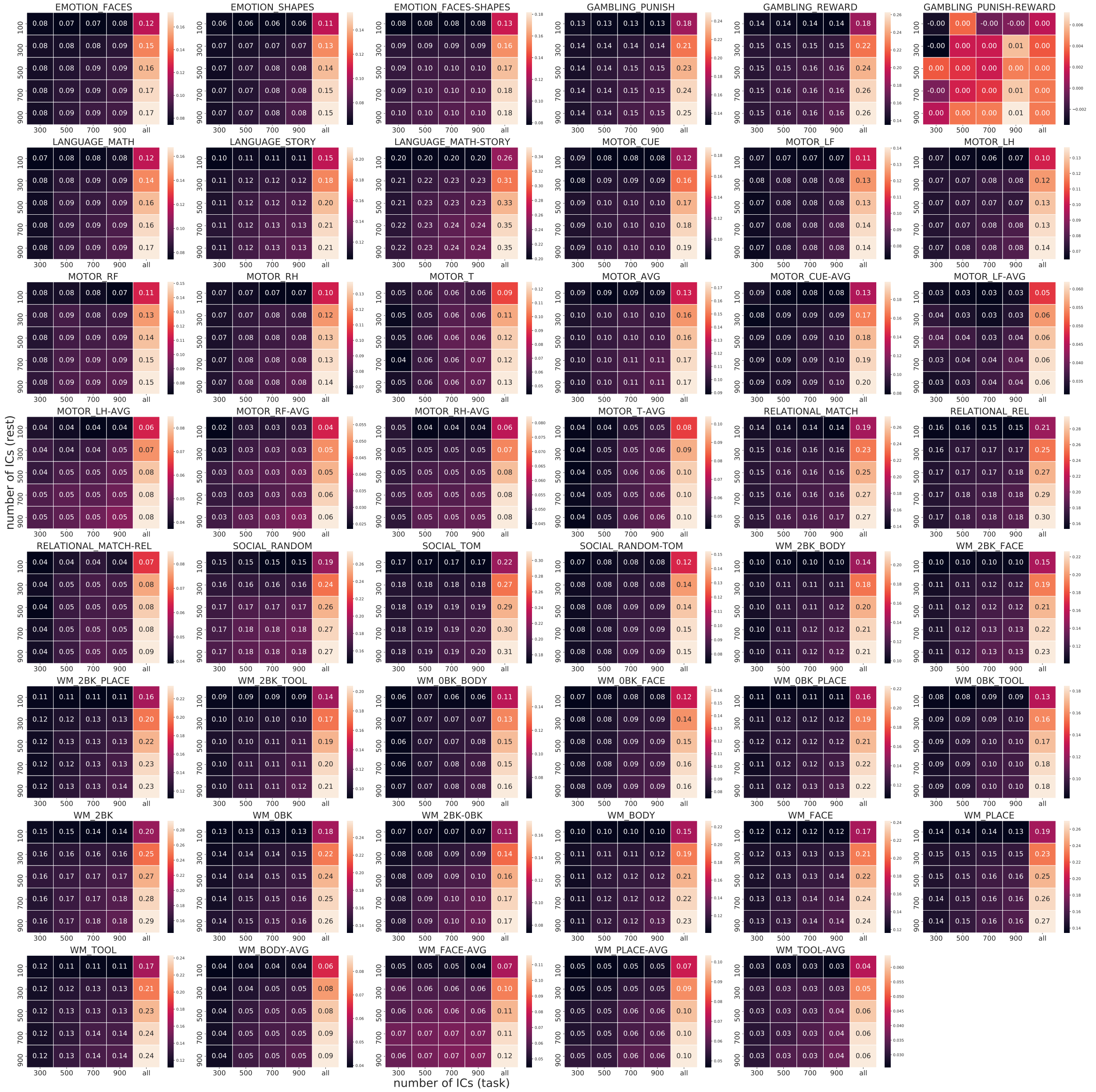

Figure S2. Prediction accuracy of the sparse model at a range of PFM dimensions, trained on 891 HCP subjects and tested on 98. Overall, accuracy increases with the number of functional modes. The un-residualised data exhibited similar accuracy patterns, though with smaller differences between the choices of dimensions (not shown here).

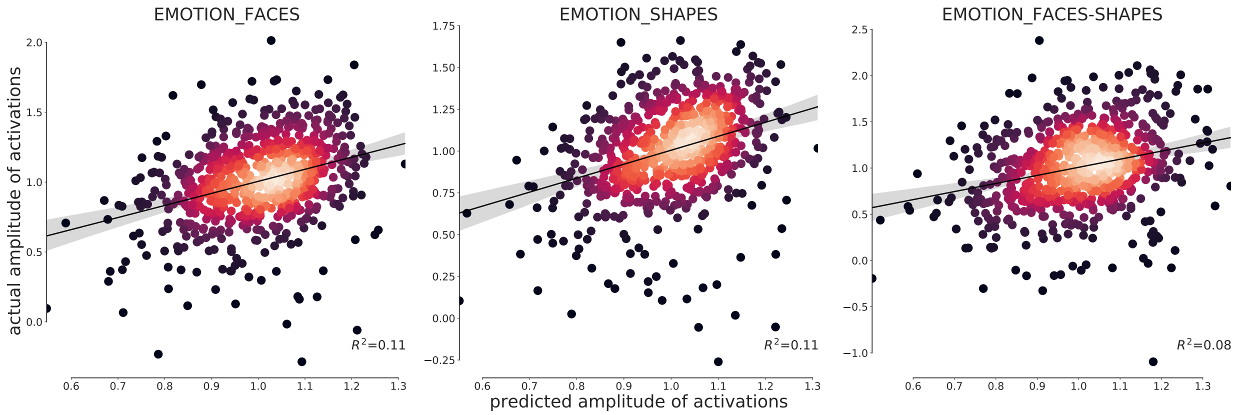

Figure S3. Using resting-state amplitude to predict activation amplitude (UKB). For each task contrast, the activation amplitude was predicted using the amplitude of the 50 PFMs (700 subjects shown).

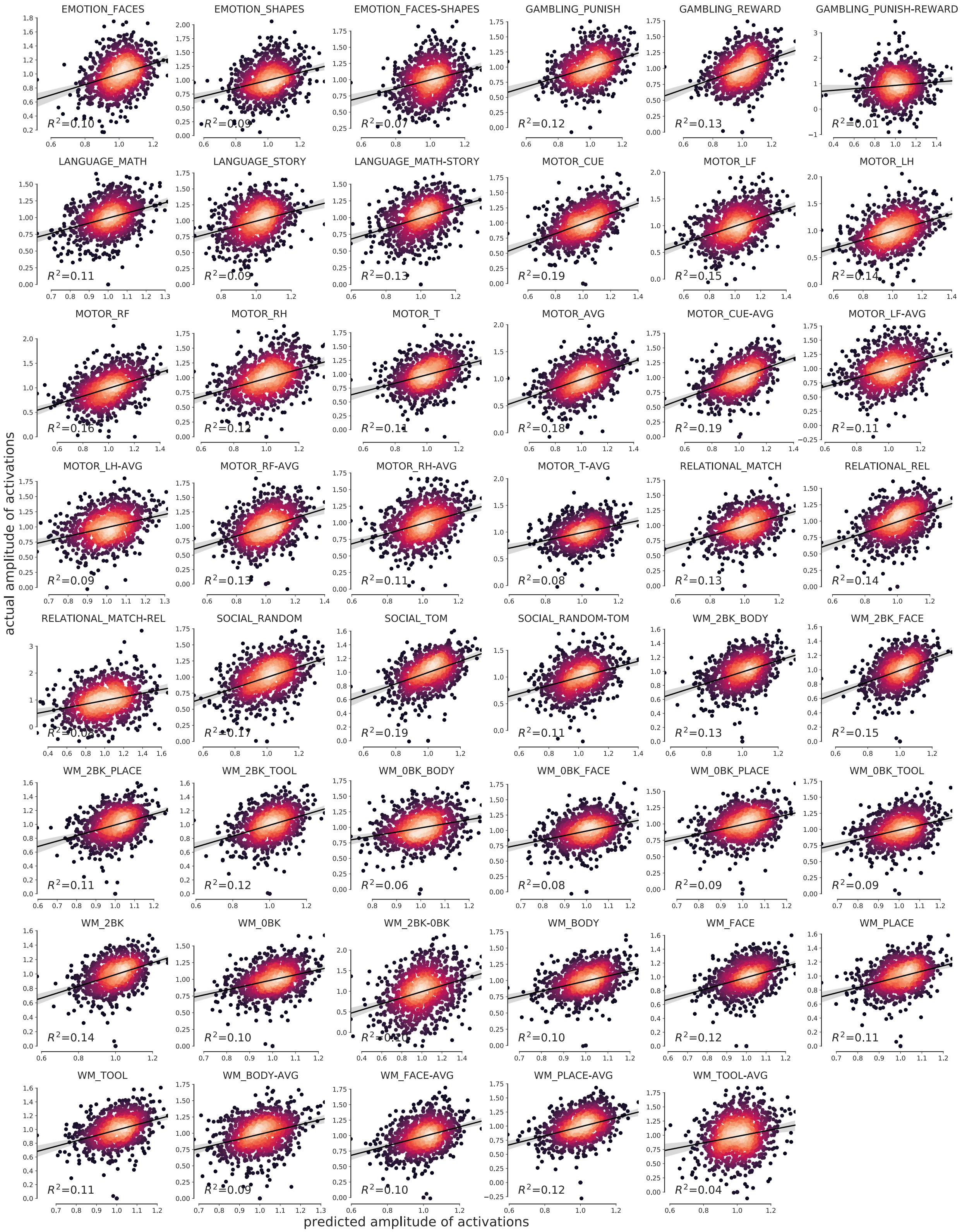

Figure S4. Using resting-state amplitude to predict activation amplitude (HCP). For each task contrast, task amplitude was predicted using the amplitude of 150 PFMs via 10-fold cross-validation (i.e., trained on 9 folds and predicted on the rest).

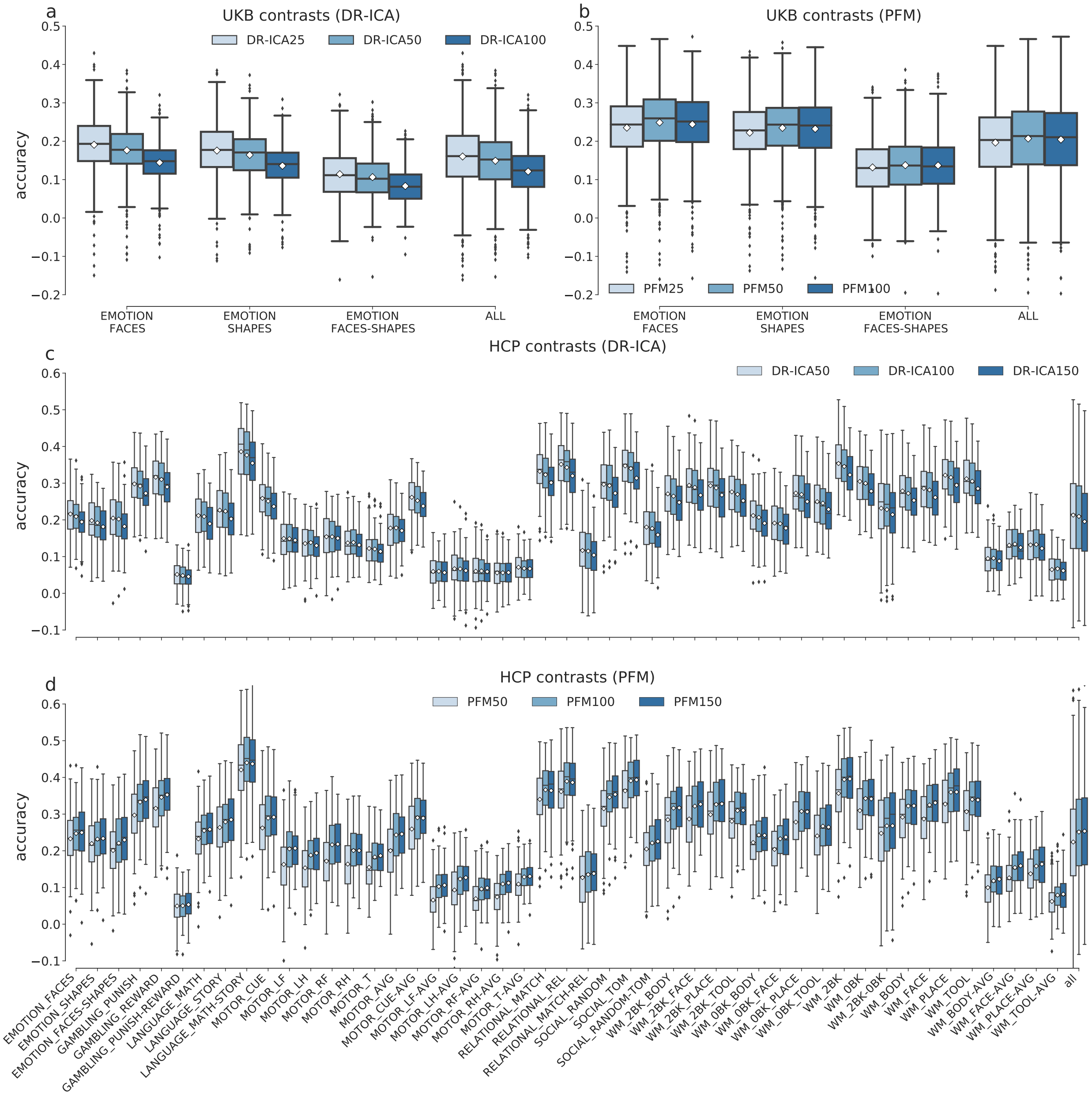

Figure S5. Choices of the functional modes' dimensions. Lower dimensions often result in larger parcels and tend to reflect whole brain resting-state networks. Higher dimensions, in contrast, tend to break down the parcels into more fine-grained functional subprocesses. We argue that the resolution of functional parcellations may non-trivially impact prediction of individual variations in task-evoked activations. The number of resting-state modes must be optimised in the first place for the the subsequent analysis. White diamond shows the mean. (a) Prediction accuracy of DR-ICA25, DR-ICA50, and DR-ICA100 across a subset of 700 UKB subjects. (b) Equivalent plots of PFM25, PFM50, and PFM100. (c) and (d) Equivalent plots of 98 HCP subjects at 50, 100, and 150 modes. The results were based on the residualised data; for the un-residualised data, different dimensions had similar effects on the accuracy, though with smaller differences (not shown here).

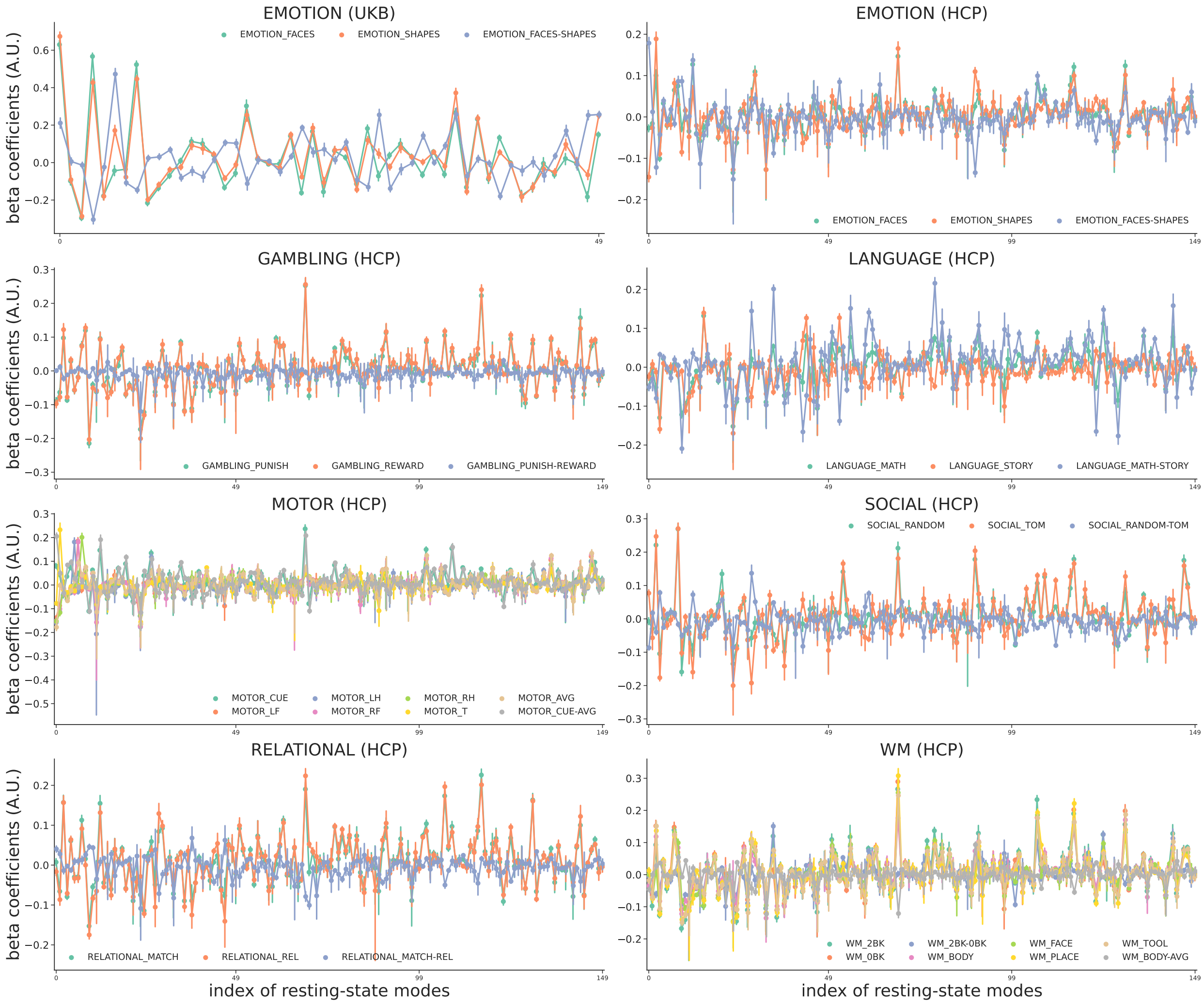

Figure S6. Baseline coefficients (betas of the baseline model) of the residualised data. Error bars showing 95% CI of the mean beta values (calculated across 1,000 UKB subjects and 891 HCP subjects). For each subject, the coefficients were divided by the maximum beta value within the given contrast. Overall, the non-differential functional modes exhibited consistent patterns within each task domain, while the differential contrasts showed different patterns.

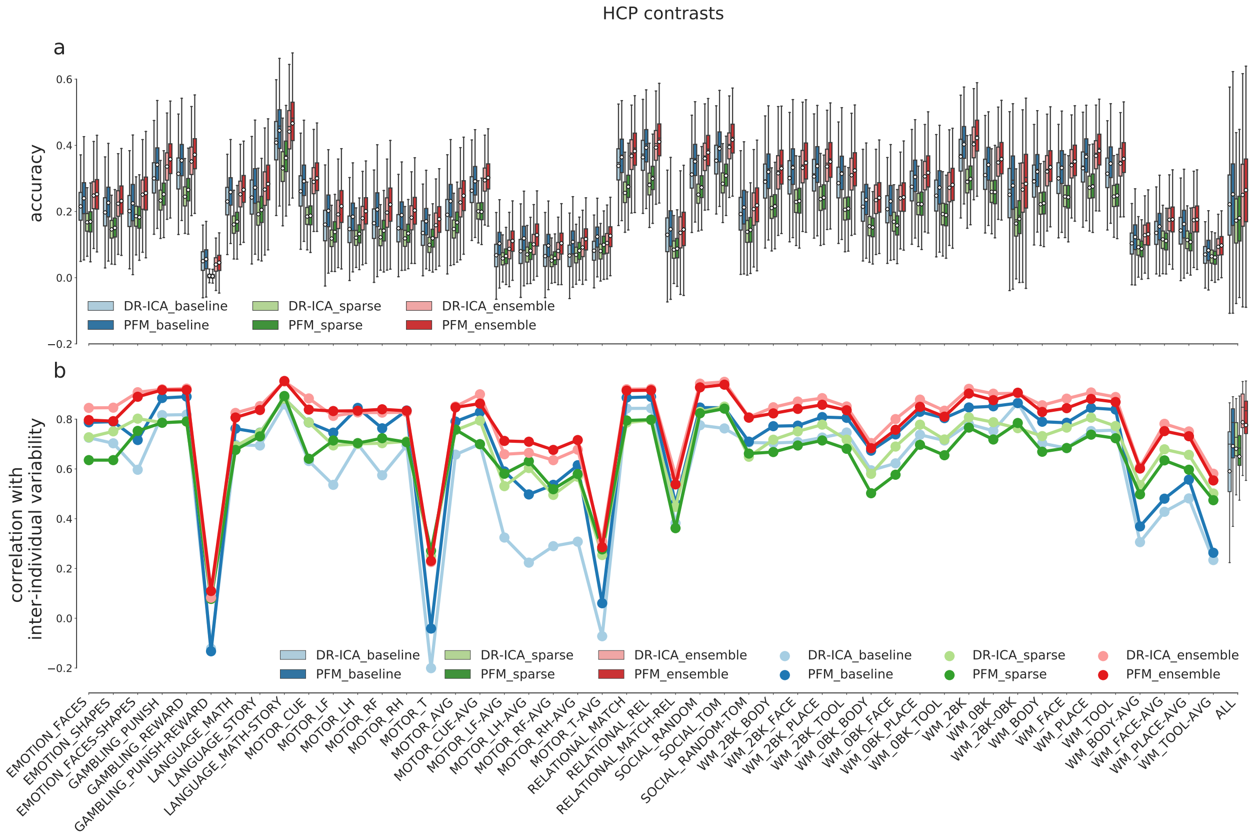

Figure S7. Comparison between the ensemble model and the single models, shown across all 47 HCP task contrasts. (a) Equivalent plots of Figure 2b. (b) Equivalent plots of Figure 2d.

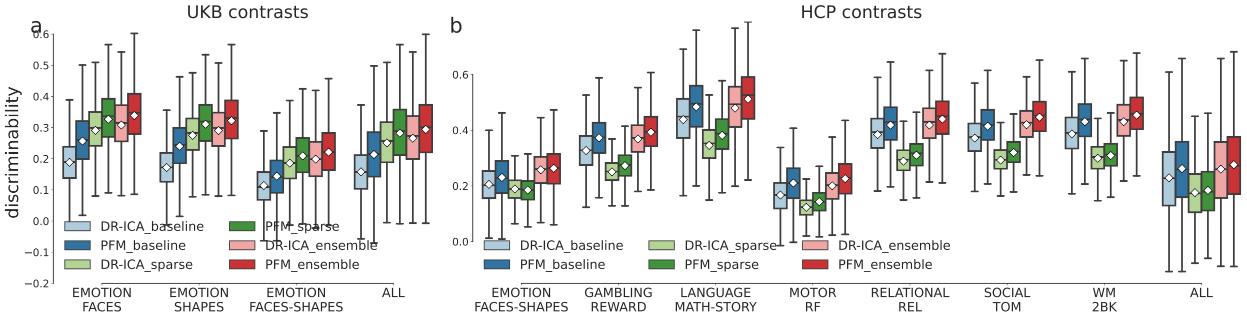

Figure S8. Discrimination metric for the residualised contrast maps. **(a)** and **(b)**: equivalent plots of Figure 2a and 2b., showing discriminability for the same sets of subjects. The discriminability values are close to the accuracy (correlation) values, because the "null models" mostly have zero accuracy.

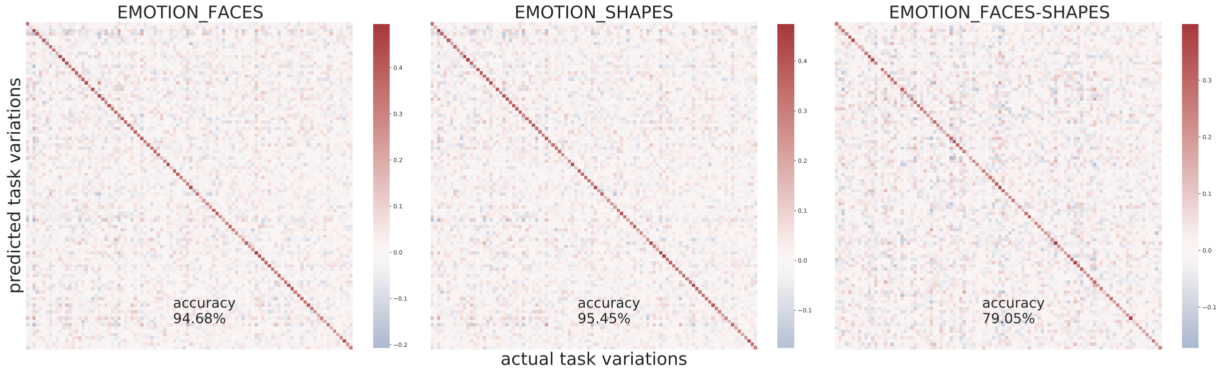

Figure S9. UKB subjects identification accuracy (based on residualised data). Accuracy (pearson's correlation between the predicted and actual variations) was calculated across all subjects; for illustration purpose, only 100 subjects were shown above. The off-diagonal elements fluctuate around zero, i.e., accuracy and discriminability calculated on residualised predictions are almost identical.

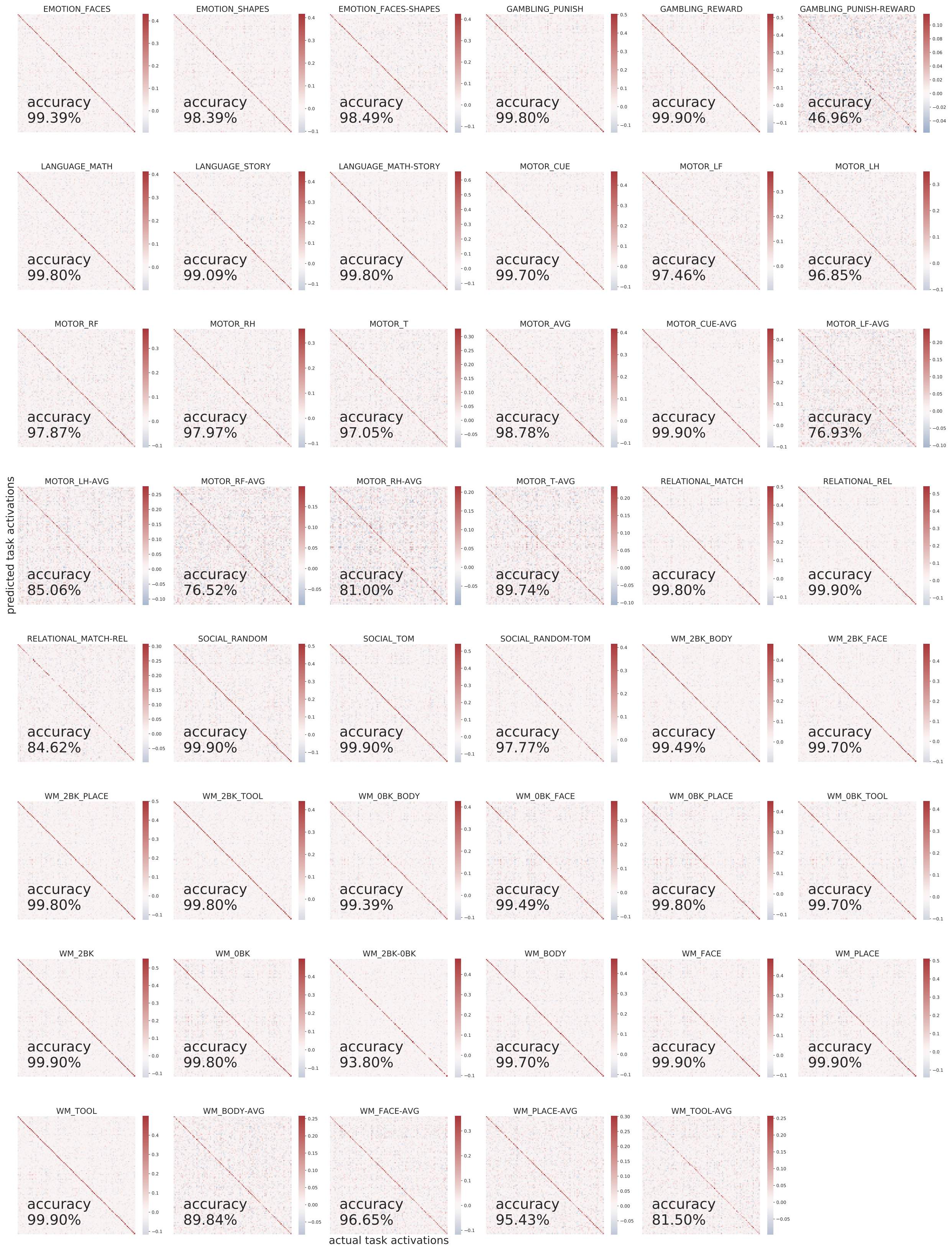

Figure S10. HCP subjects identification accuracy (based on residualised data). For illustration purpose, only 100 subjects were shown above.

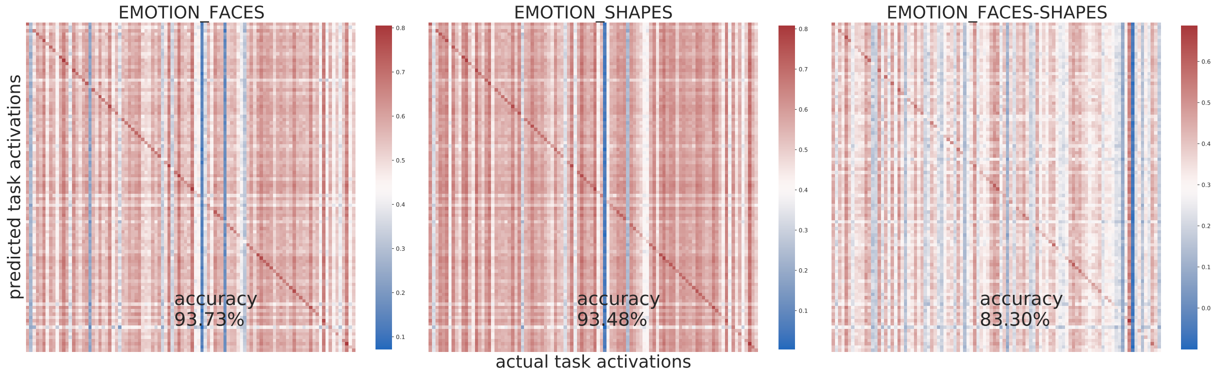

Figure S11. UKB subjects identification accuracy (with group-average activations added back in). The off-diagonal values no longer fluctuate around zero. The subject identification accuracy remains high. For illustration purpose, only 100 subjects were shown above.

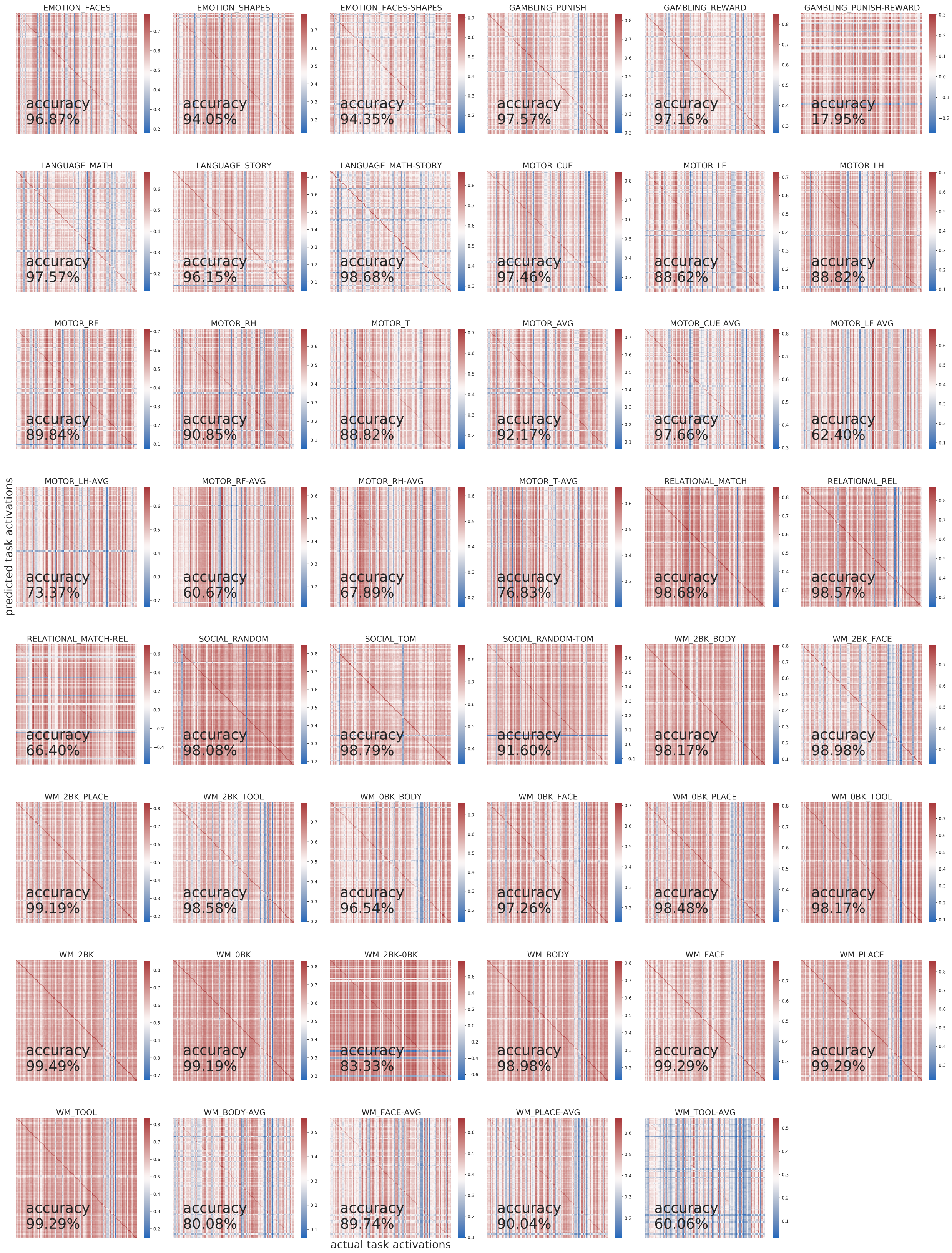

Figure S12. HCP subjects identification accuracy (with group-average activations added back in). For illustration purpose, only 100 subjects were shown above.

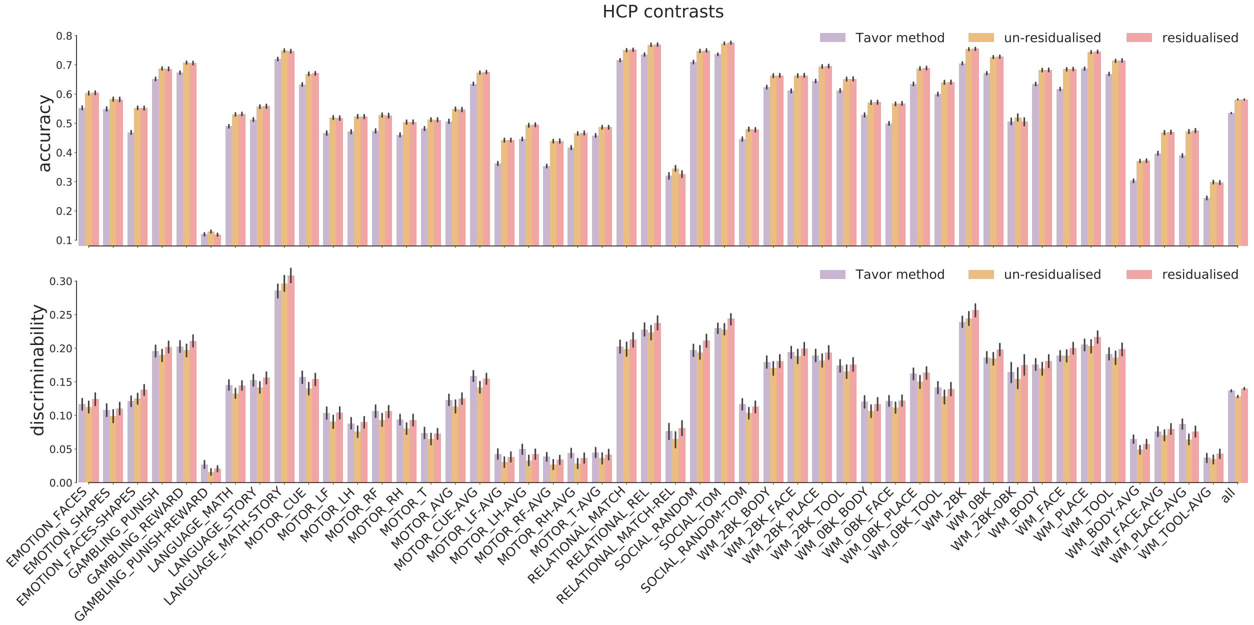

Figure S13. Comparison of the Tavor model and the ensemble model (both un-residualised and residualised) across 991 HCP subjects for all 47 task contrasts. The ensemble model (either residualised or not) outperformed the Tavor model in terms of the actual prediction accuracy; however, the Tavor model could make more individualised predictions than the ensemble model if both trained on un-residualised data. The residualised ensemble model outperformed the other two both in accuracy and discriminability, except for the motor task domain.

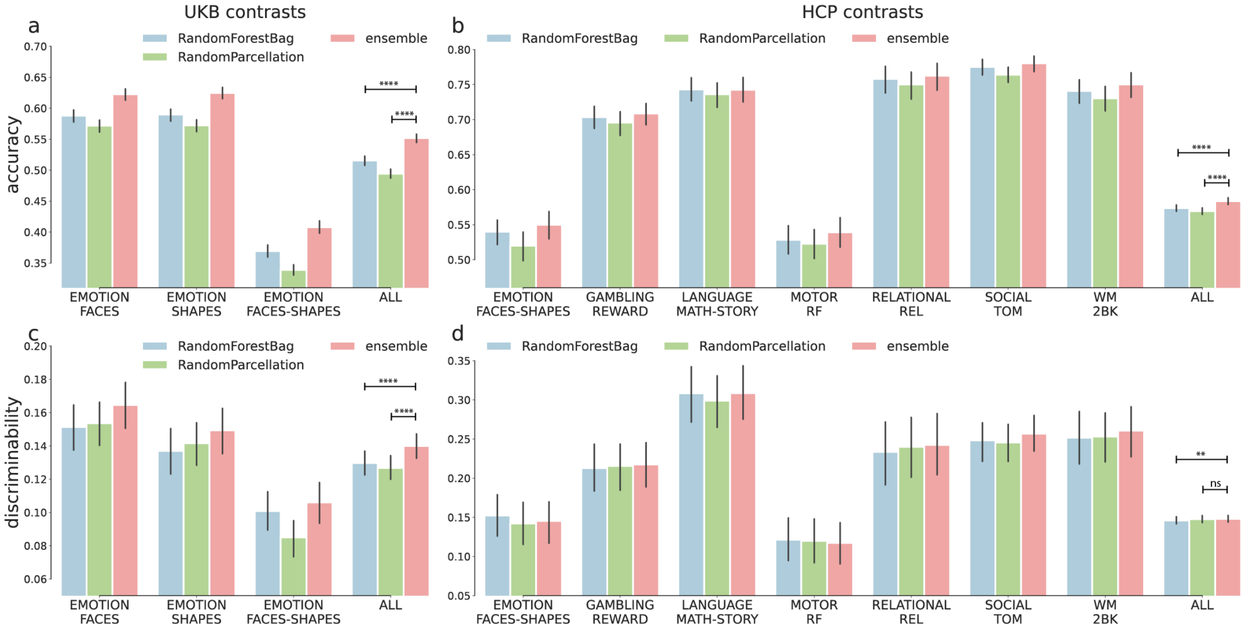

Figure S14. Prediction accuracy and discriminability of the three methods. The hyper-parameters of Random Forest Bagging were optimised on a different subset. P-values of the two-sided paired t-tests are Bonferroni-corrected (8 tests in total). ns: P > 0.05; **: P < 0.01; ****: P < 0.0001. Blue: the Random Forest Bagging approach used in Cohen et al., 2020; Green: the Random Parcellation approach used in Dohmatob et al. 2021; Red: Our ensemble approach. (a) Prediction accuracy across 700 UKB subjects, trained on 4,000 subjects. (b) Prediction accuracy across 98 HCP subjects, trained on 991 subjects. (c) Equivalent plot of (a), showing prediction discriminability. (d) Equivalent plot of (b), showing prediction discriminability.

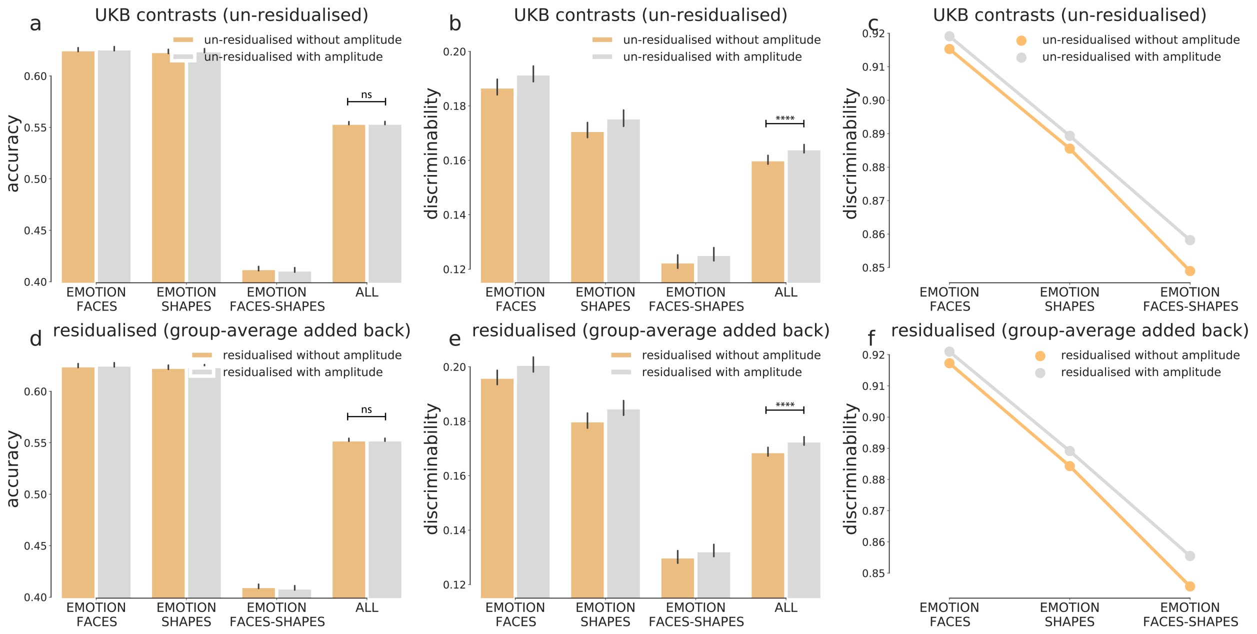

Figure S15. Prediction accuracy and discrimininability of the ensemble model with or without PFM amplitude as additional features, calculated across 17,560 UKB subjects Although incorporating amplitude did not further increase the overall accuracy for UKB, it did marginally improve prediction discriminability. This coincides with (c) and (d), which shows that the std. maps of predicted activations (across subjects) exhibited higher correspondence with the actual inter-individual variability. P-values of the two-sided paired t-tests are Bonferroni-corrected (4 tests in total). ns: P > 0.05; **: P < 0.01; ****: P < 0.0001.

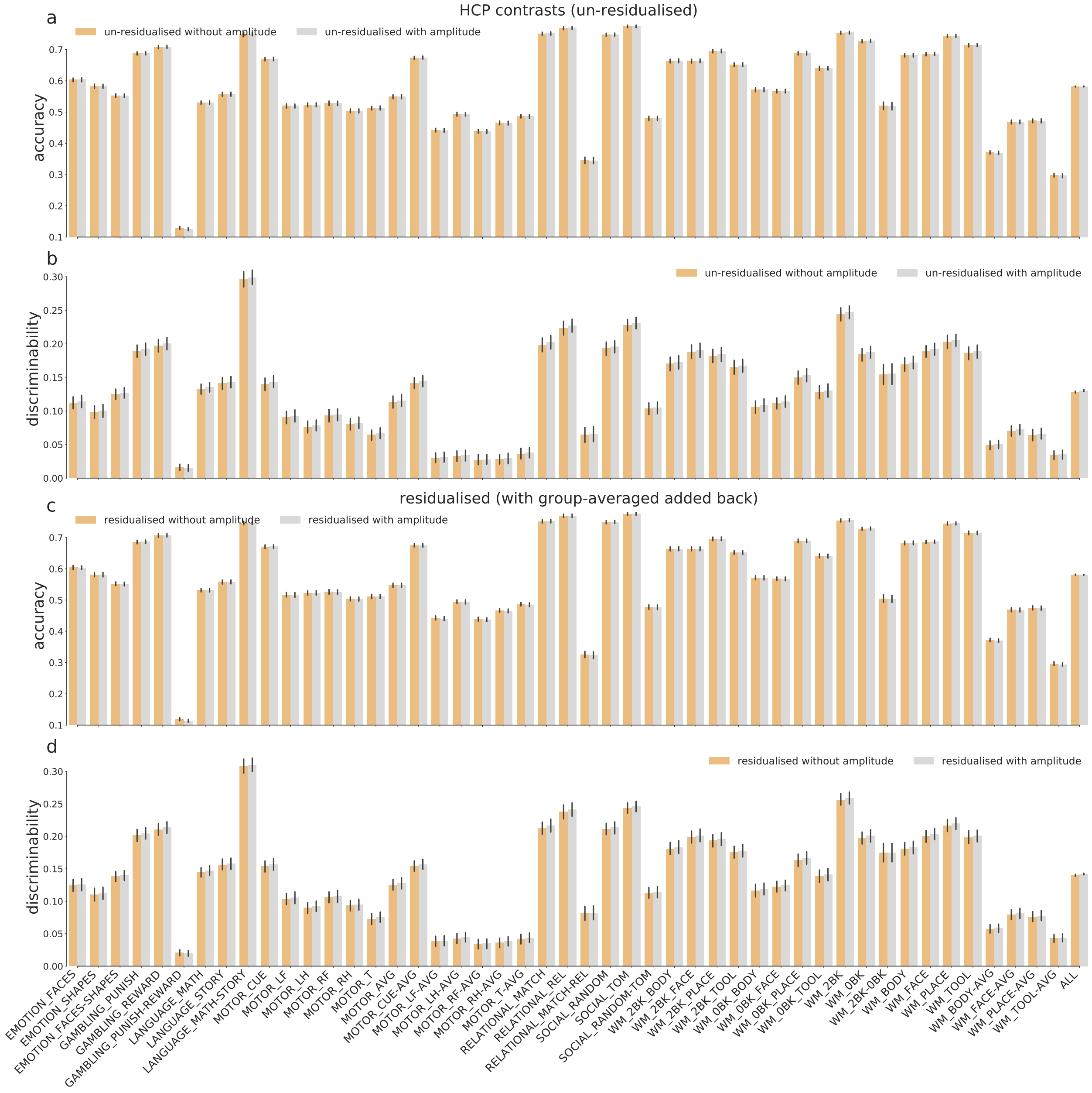

Figure S16. Prediction accuracy and discrimininability of the ensemble model with and without resting-state amplitude as additional features for all 47 HCP contrasts. For HCP, however, including PFM amplitude as additional features at the ensemble stage only marginally improved prediction discriminability.

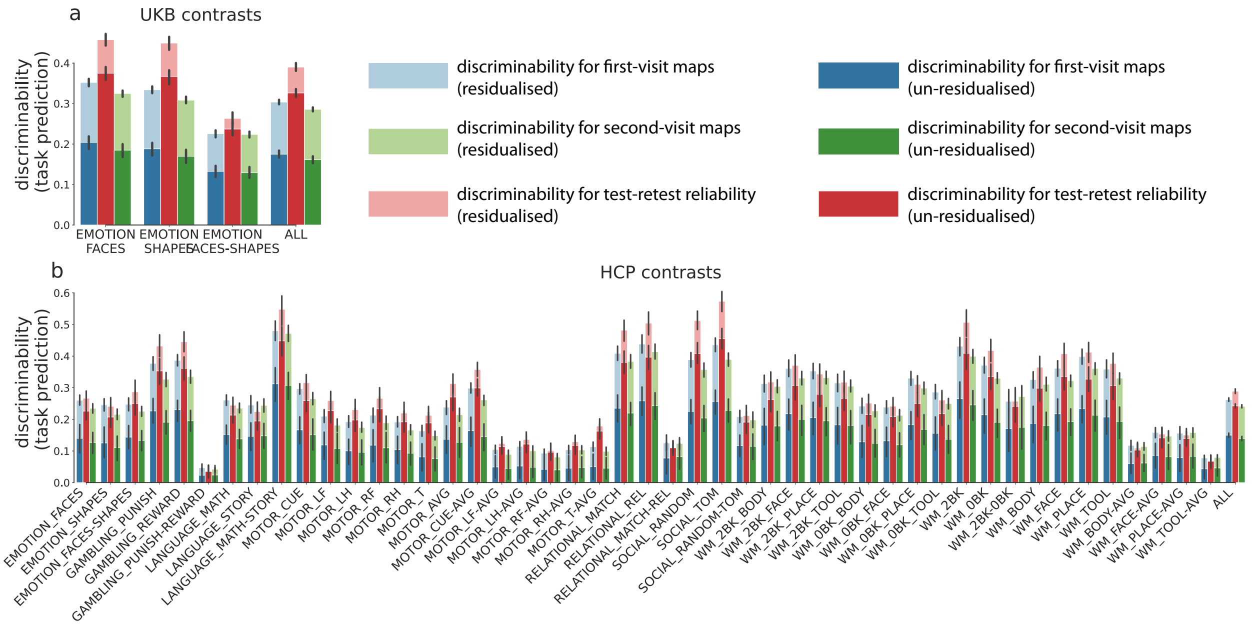

Figure S17. Discrimination metric for the predictions and the test-retest reliability. Blue: prediction discriminability for the first-visit task contrast maps. Green: prediction discriminability for the second-visit task contrast maps. Red: test-retest discriminability of task contrast maps. Note that opposite to Figure 4b and 4c, here light colours denote the prediction discriminability of the residualised maps, dark colours the discriminability of the un-residualised maps.

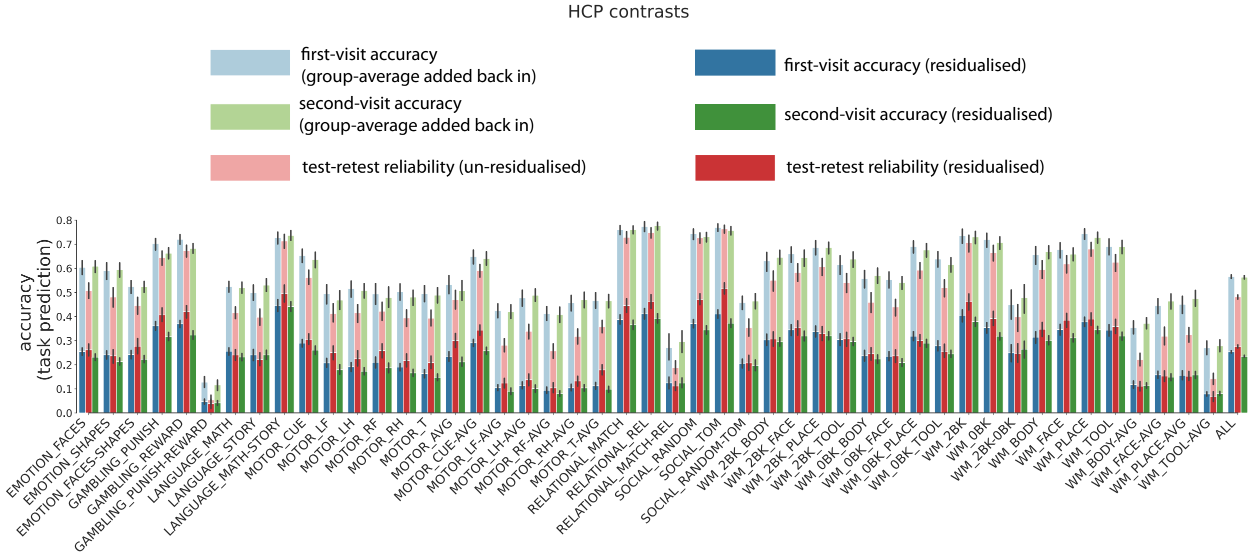

Figure S18. Prediction accuracy of first-visit and second-visit contrast maps and test-retest reliability for all HCP contrasts. Equivalent plots of Figure 4c, for all HCP task contrasts.

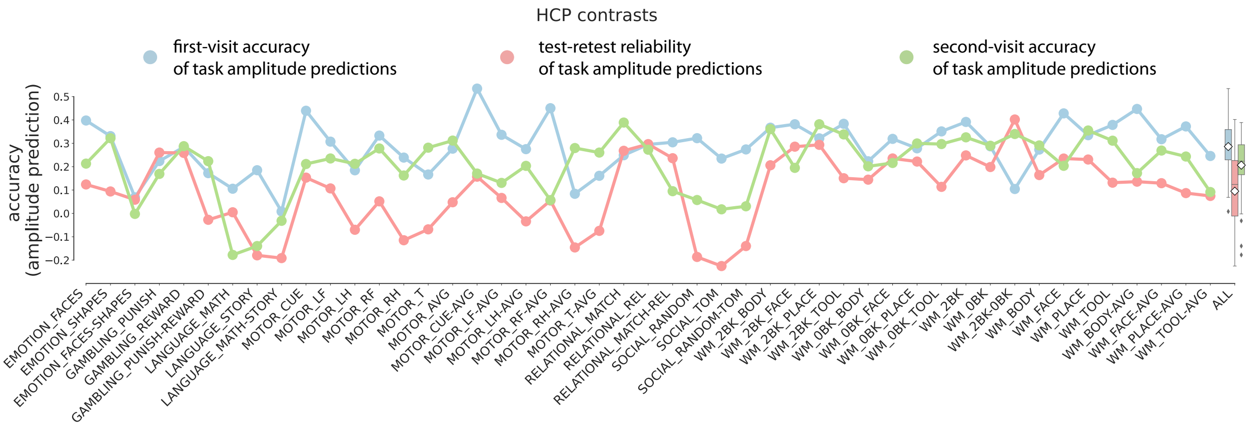

Figure S19. Prediction accuracy of first-visit and second-visit amplitude and test-retest reliability for all HCP contrasts. Equivalent plots of Figure 4e, for all HCP task contrasts.

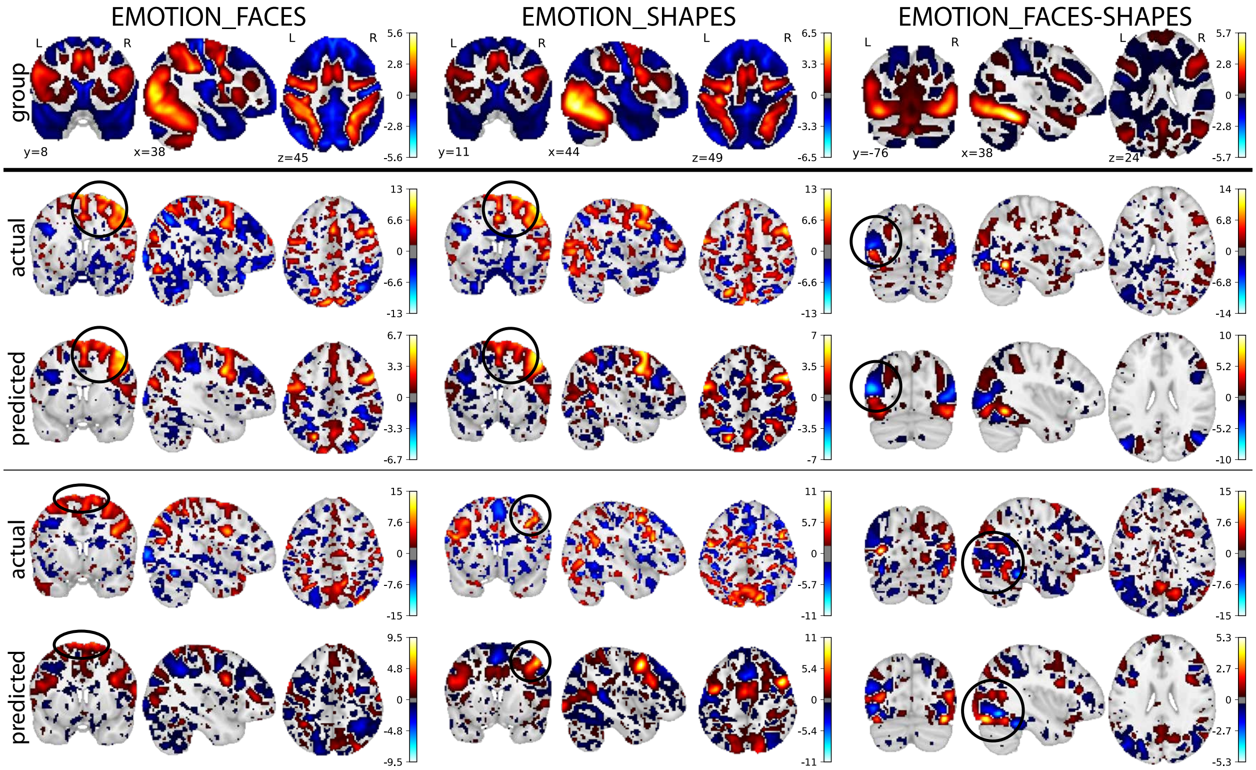

Figure S20. The actual and the predicted task variations (residuals) of the example UKB subjects, shown on the brain.

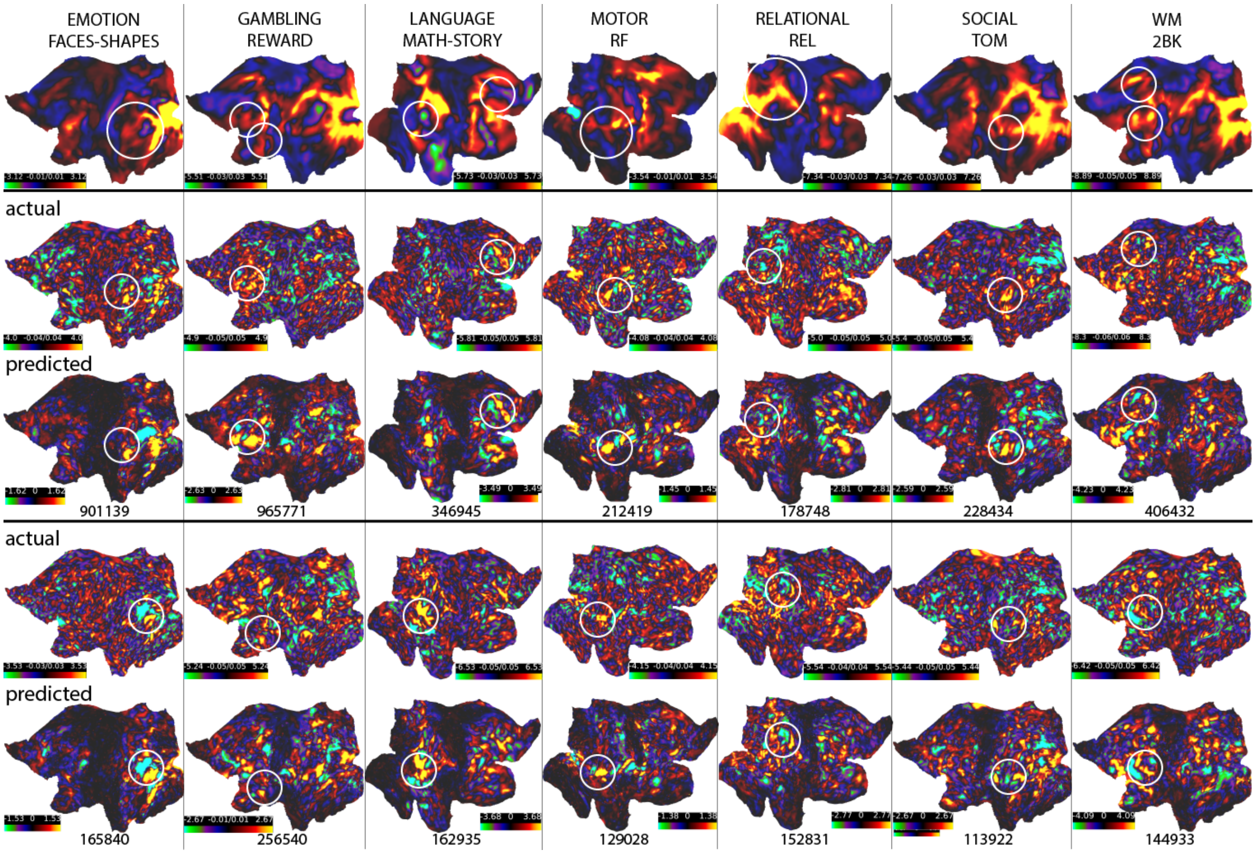

Figure S21. The actual and the predicted task variations (residuals) of the HCP subjects, shown on the surface.

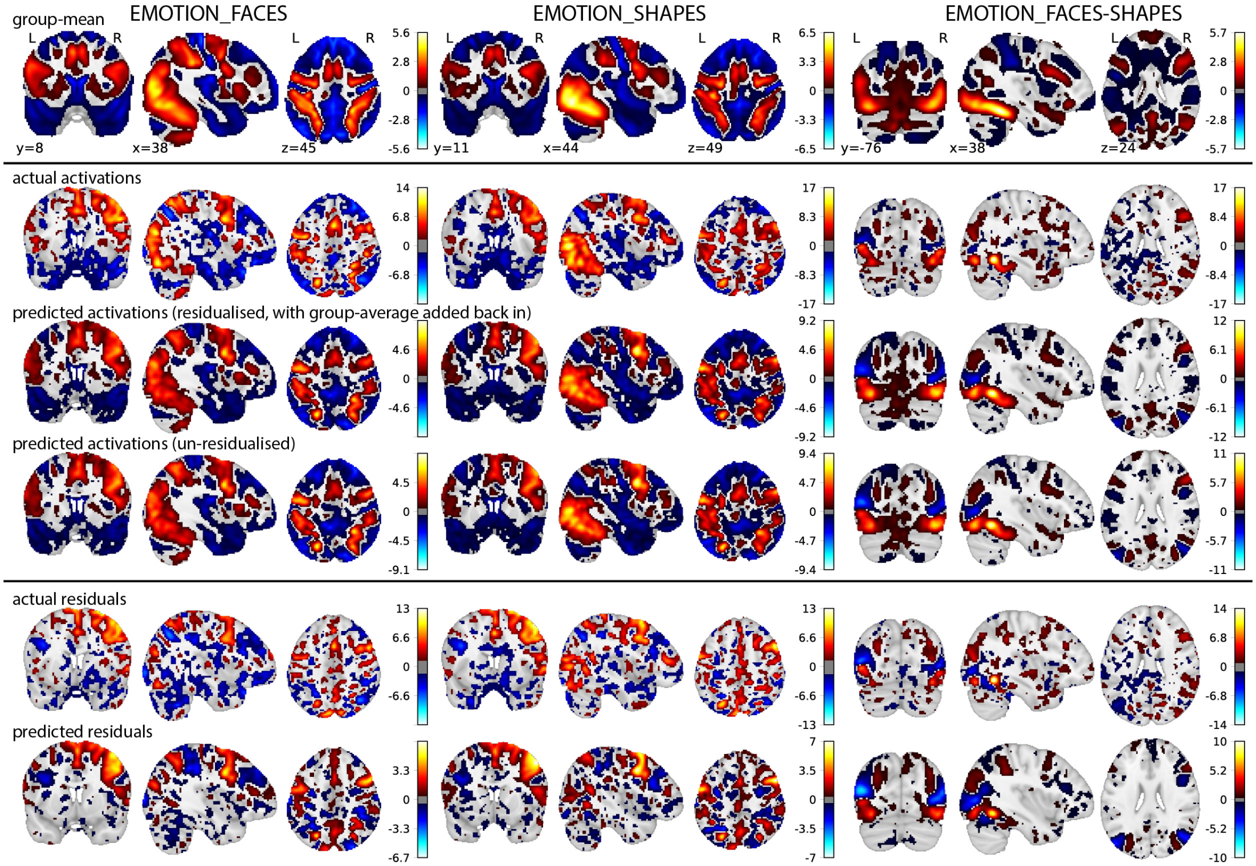

Figure S22. UKB subjects. We show the comparisons between 1) the group-average; 2) the actual activations; 3) the predicted activations (residualised, with group-average added back in); 4) the predicted activations (un-residualised); 5) the actual residual maps; 6) the predicted residual maps, one example subject per contrast. The predicted residual contrast maps with group-average added back in looked visually identical to the un-residualised predicted task contrast maps. This is unsurprising considering that both gave high and close discriminability values. Furthermore, the group-average added back in to the predicted residuals was scaled by the resting-state-predicted amplitude, which was only a noisy estimate of the overall activation amplitude. Thus, with the group-average added back in, the accuracy and discriminability of residual predictions might be compromised. Although the un-residualised predictions looked almost identical to those residualised ones with the group-average added back in on the chosen example subjects, we would like to point out that statistically, the residualised approach gave significantly better individualised predictions, across the pools of subjects, Bonferroni-corrected.

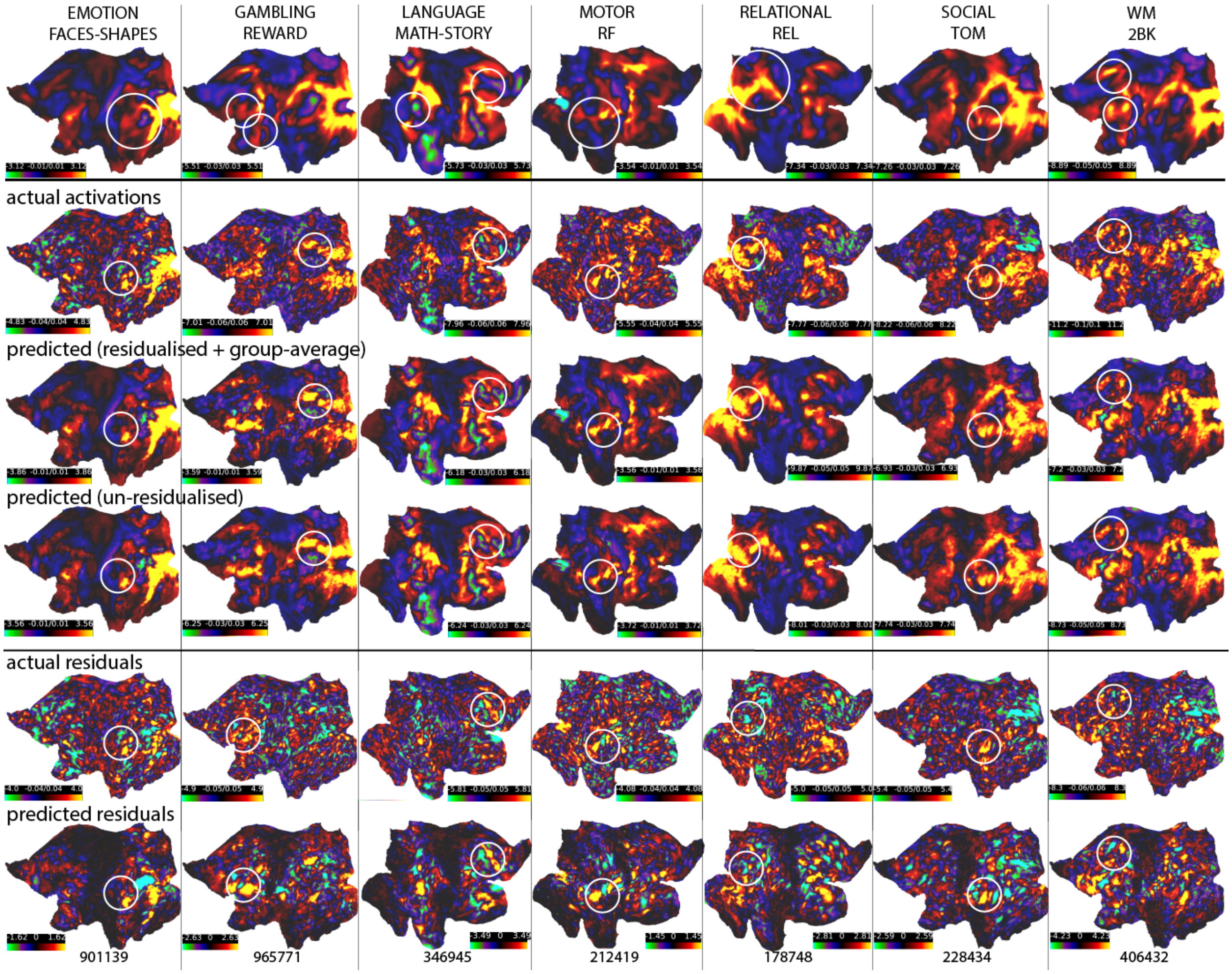

Figure S23. HCP subjects. We show the comparisons between 1) the group-average; 2) the actual activations; 3) the predicted activations (residualised, with group-average added back in); 4) the predicted activations (un-residualised); 5) the actual residual maps; 6) the predicted residual maps, one example subject per contrast.

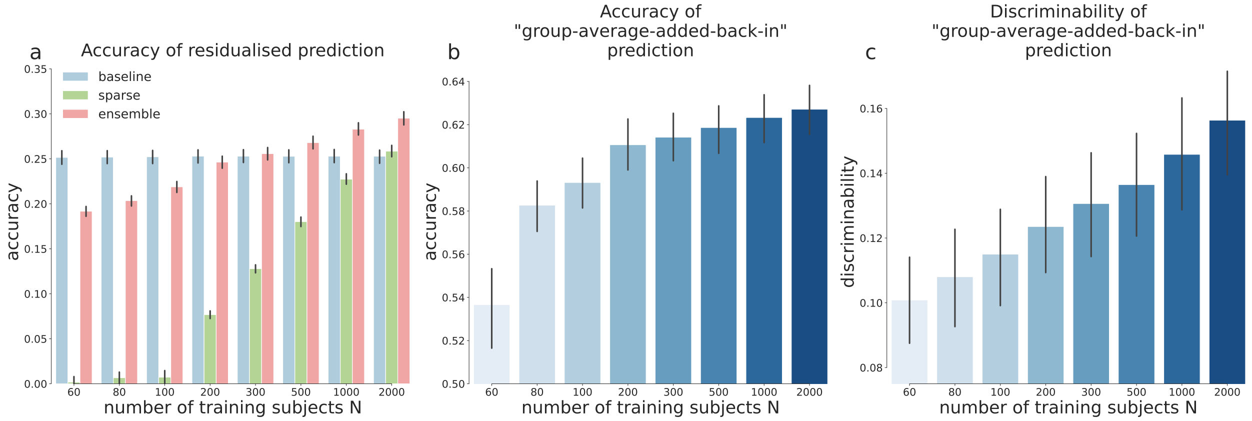

Figure S24. Prediction accuracy as a function of number of training subjects (UKB). (a) Prediction accuracy of the baseline, sparse and ensemble model for the residualised maps. The baseline and ensemble model converges very quickly; the sparse model requires a larger number of training subjects. (b) Prediction accuracy of the ensemble model, with group-average added back in. (c) Equivalent plot of (b), showing prediction discriminability.

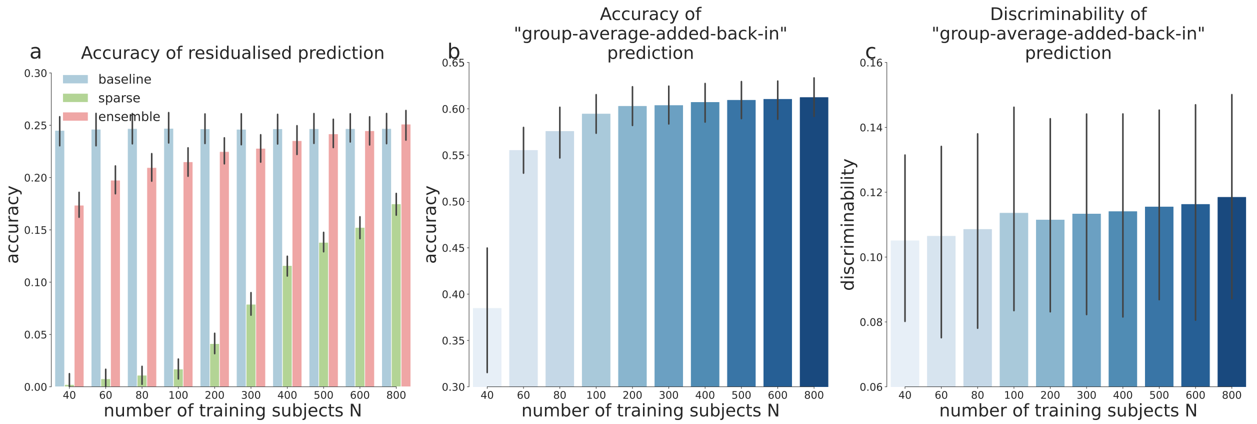

Figure S25. Prediction accuracy as a function of number of training subjects (HCP). (a) Prediction accuracy of the baseline, sparse and ensemble model on the residualised maps for 98 HCP subjects. The baseline and ensemble model gave good accuracy at a small N; the sparse model requires a larger number of training subjects. (b) Prediction accuracy of the ensemble model, with group-average added back in. (c) Equivalent plot of (b), showing prediction discriminability.
